# Service Life of Adhesive Bonds under Cyclic Loading with a Filler Based on Natural Waste from Coconut Oil Production

**DOI:** 10.3390/polym14051033

**Published:** 2022-03-04

**Authors:** Petr Hrabě, Viktor Kolář, Miroslav Müller, Monika Hromasová

**Affiliations:** 1 Department of Material Science and Manufacturing Technology, Faculty of Engineering, Czech University of Life Sciences Prague, Kamycka 129, 165 00 Prague-Suchdol, Czech Republic; hrabe@tf.czu.cz (P.H.); muller@tf.czu.cz (M.M.); 2 Department of Electrical Engineering and Automation, Faculty of Engineering, Czech University of Life Sciences Prague, Kamycka 129, 165 00 Prague-Suchdol, Czech Republic; hromasova@tf.czu.cz

**Keywords:** coconut shell powder, natural filler, material utilization, secondary product, mechanical properties, quasi-static test, cracking, SEM

## Abstract

The research is focused on the evaluation of mechanical properties of adhesive bonds with a composite layer of adhesive to increase their service life (safety) under cyclic loading of different intensities. Cyclic loading represents a frequent cause of adhesive bond failure and, thus, a reduction in their service life. Waste from the production of coconut oil, that is, coconut shells in the form of particles, was used as a filler. Coconut shells are in most cases incinerated or otherwise uselessly incinerated, but they can also be used as a natural filler. Cyclic loading (quasi-static tests) was performed for 1000 cycles in two intensities, that is, 5–30% (157–940 N) of maximum force and 5–50% (157–1567 N) of maximum force. The results of the experiment showed a positive effect of the added filler, especially at an intensity of 5–50%, when the service life of adhesive bonds with a composite adhesive layer (AB10, AB20, AB30) increased compared to adhesive bonds without added AB0 filler, which did not withstand the given intensity. A more pronounced viscoelastic behavior of adhesive bonds was demonstrated at an intensity of 5–50% between the 1st and 1000th cycle. SEM analysis showed reduced wetting of the filler and matrix and delamination due to cyclic loading.

## 1. Introduction

Adhesive bonding technology is currently one of the promising methods of bonding structural elements. Bonding has several advantages, such as the production of corrosion-resistant, light, and rigid bonds, good vibration damping properties or the ability to bond materials in various industries [1,2,3,4]. Adhesive bonding provides not only a connecting function, but also a supporting function, that is, sealing, clamping, and securing [5,6]. Another advantage of adhesive bonds is the wide range of modifications of adhesives using different reinforcing phases in the form of particles or short and long fibers of synthetic or natural origin [7].

Waste, and its production, is a global topic. Natural waste, e.g., from post-harvest lines of agricultural commodities, etc., is largely incinerated or otherwise disposed of, but reuse of all materials generated during the processing of agricultural crops can increase the economic efficiency of the whole process and reduce the negative impact on the environment [8]. The material use of natural waste as a filler indicates the current research trend in the field of research of composite materials with a matrix based on polymer material. A number of prestigious workplaces or scientific journals deal with the application of natural materials (waste) in specific applications of world industry [9]. Of course, there is an alternative in the form of composting, etc., but natural waste can be used in other ways, such as filler [10]. By modifying the adhesives with a natural particulate filler, a composite layer can be obtained, which can improve the parameters of the adhesive bond in terms of mechanical properties [11]. Many authors have dealt with the use of various types of shells as fillers in the field of polymer composites [12,13,14]. Keerthika et al. [15] state that the coconut shell filler is used due to its high carbon content and hardness. Orue et al. [16] used walnut shell as a filler in PLA to investigate its mechanical properties. Ramnath et al. [17] used sea shell as a particulate filler in the polymer matrix to investigate the mechanical properties of the resulting polymer composite.

The aim of several research institutes is to increase the efficiency of the use of adhesive bonds in practice. The main experimental program is to increase the strength of adhesive bonds [18,19,20]. Physical and chemical factors (wettability, adhesion and cohesion, aging, degradation environment) [5,21,22], technological factors (roughness and structure of the bonded surface or filler) [23,24,25], and design factors (size of bonded area and type of load applied) affect the strength of adhesive bonds [26,27]. The resulting properties of the adhesive bond are given by the synergy of the mentioned factors.

In practice, adhesive bonds are exposed to many complicated loading conditions, such as polymer matrix cracking, etc. Cracking is a phenomenon of gradual accumulation of plastic deformation when materials and structures are exposed to cyclic loading with non-zero medium stress. The accumulation of plastic deformation is an important aspect of fatigue damage [28,29]. Under monotonic loading conditions, polymeric materials can withstand a higher stress level compared to alternating loads. As a result of the periodic nature of the applied load, micro-cracks initiate and propagate at relatively low stress levels and finally the structure will fracture [30]. For polymeric materials, that is, for adhesive bonds in the adhesive layer, the response to cyclic loading is primarily viscoelastic, but in the case of higher values of cyclic loading, cracking can also occur [31]. The viscoelastic response to cyclic loading is given mainly by the medium stress and its amplitude [32]. Therefore, cracking is one of the key factors in the design of structural elements, which should be considered when applying adhesive bonds. A better understanding of fatigue behavior is essential for the application of adhesive bonds in practice [28,29].

The aim of the research was to evaluate the mechanical properties of adhesive bonds with a composite layer of adhesive with a filler based on coconut shell particles under cyclic loading with different loading intensities, and the related evaluation of their service life (safety). Cyclic loading is a common cause of bond failure and, therefore, negatively affects its service life.

## 2. Materials and Methods

### 2.1. Materials

#### 2.1.1. Filler

**Figure 1:**
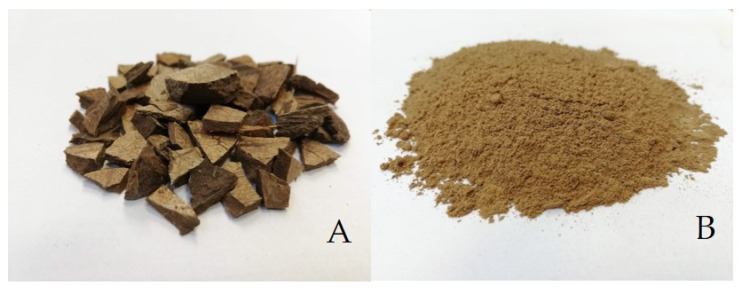
Waste from coconut oil production: (**A**): pre-crushed coconut shells, (**B**): natural particulate filler from coconut shells.

**Figure 2:**
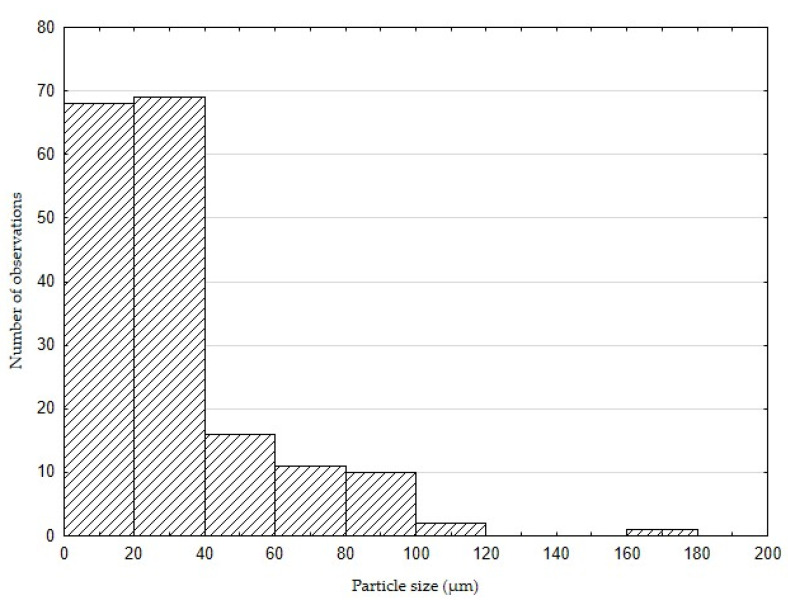
Histogram of coconut shell filler particle sizes.

Figure 1 shows waste from the production of coconut oil, that is, coconut shells. Coconut shells were crushed to approximately 1 cm (Figure 1A) and dried in a laboratory oven at 105 °C for 24 h. The shells were then crushed with Retsch MM 400 oscillating mill (Retsch Verder s.r.o., Prague, Czech Republic) and sorted into individual fractions by sieve analysis. The resulting fraction size was determined using a 100 μm mesh screen. The average particle size was 55 ± 35 μm. Figure 1B presents the final form of filler used to make the composite adhesive layer.The size of the particulate filler was measured with Gwyddion program (version 2.49, David Nečas and Petr Klapetek, Brno University of Technology, Brno, Czech Republic). The histogram of the particle size frequency is shown in Figure 2. Coconut shell contains approximately 33 wt.% lignin, 27 wt.% cellulose, 31 wt.% hemicellulose, and 0.6 wt.% ash [33,34].

#### 2.1.2. Matrix and Bonded Material (Adherent)

The matrix was a two-component epoxy resin Epoxy 1200 (CHS-Epoxy 324) (Havel Composites CZ s. r. o., Svésedlice, Czech Republic) with hardener P11 (Havel Composites CZ s. r. o., Svésedlice, Czech Republic). The weight ratio of resin to hardener was 100:7.

**Figure 3:**
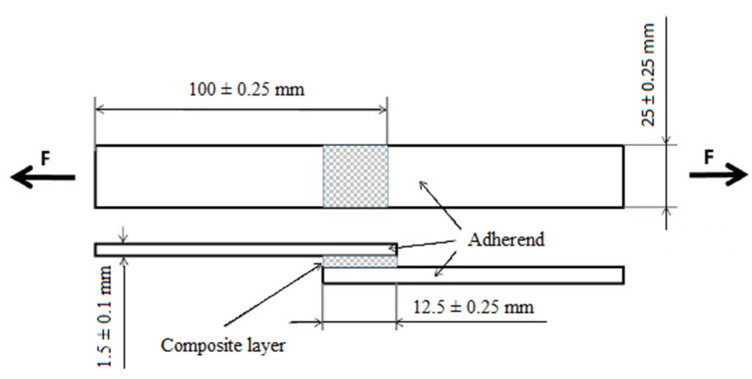
Scheme of the adhesive bond according to ČSN EN 1465 [36].

Structural carbon steel S235J0 (Ferona a.s., Prague, Czech Republic) with a thickness of 1.5 ± 0.1 mm, a length of 100 ± 0.25 mm, and a width of 25 ± 0.25 mm was used as adherend. The basic mechanical properties of the steel used are given in Table 1. The dimensions of the adherent were determined according to the ČSN EN 1465 standard [35]. Figure 3 presents a diagram of adhesive bonds, including their dimensions according to the standard. The adherents were mechanically and chemically treated shortly before application of the composite layer. The mechanical surface treatment of the adherents was carried out in a blasting chamber with abrasive Garnet MESH 80, and the chemical surface treatment was carried out in an acetone bath. The surface of the treated adherents was subjected to roughness measurement with a Mitutoyo Surftest 301 profilometer (Mitutoyo Europe GmbH, Neuss, Germany), where Ra = 3.48 ± 0.21 μm and Rz = 11.34 ± 0.28 μm.

#### 2.1.3. Production and Types of Adhesive Bonds

The production of adhesive bonds was carried out according to the ČSN EN 1465 standard. The adhesive was applied to the surface of the adherents in an even layer. The filler was dried in a laboratory oven at 105 °C for 3 h shortly before mixing with the matrix. Subsequently, the adherents were overlapped according to the above standard, that is 12.5 ± 0.25 mm, and loaded with a weight of 750 g (7.4 N) and left to cure the adhesive layer at a laboratory temperature of 22 ± 1 °C and a relative humidity of 41 ± 4% for 24 h. Table 2 shows the types of adhesive bonds, the thickness of the adhesive layer, and their characteristics.

### 2.2. Methods

Testing of mechanical properties was performed on a universal testing machine LABTest 5.50 ST (LABORTECH s. r. o., Opava, Czech Republic) with AST KAF 50 kN measuring unit (LABORTECH s. r. o., Opava, Czech Republic) and the evaluation software Test & Motion (version 4.5.0.15, LABORTECH s. r. o., Opava, Czech Republic) at a laboratory temperature of 21 ± 1 °C and a relative humidity of 44 ± 4%. The methodology of testing mechanical properties under cyclic loading, that is, shear strength and strain, consisted of determining the standard value obtained in the static tensile test (ČSN EN 1465) from 7 adhesive bonds marked as AB0 at a test speed of 0.6 mm × min^−1^. The standard value corresponds to the maximum force, that is, at complete failure of the adhesive bond, and its value was 3134 N (average value of the maximum force from 7 adhesive bonds). Cyclic loading (quasi-static test) was performed for 1000 cycles at a test speed of 6 mm × min^1^ between 5% of the maximum force, that is, 5% = 157 N (lower limit) and 30, 50% of the maximum force, that is, 30% = 940 N and 50% = 1567 N (upper limit). After the completion of 1000 cycles, a static tensile test automatically followed until the complete failure of the adhesive bond at a speed of 0.6 mm × min^−1^. The static test was performed only when the 1000th cycle was completed. Otherwise, the test was terminated. The time delay at the lower and upper limits was set at 0.5 s. Each test series contained 7 test specimens.

The type of fracture surface failure was evaluated based on ISO 10365 [38].

Statistical evaluation of the performed experiments was performed by analysis of variance, that is, ANOVA F-test in the program STATISTICA (version 14.0.0.15, StatSoft CR, Prague, Czech Republic). The statistical dependence on the significance level 0.05 between the adhesive bond AB0 and AB10, AB20 and AB30 was evaluated, when the hypothesis H_0_ was established, presenting a statistically insignificant difference between AB0 and AB10, AB20, and AB30 (*p* > 0.05). Hypothesis H_1_ rejects hypothesis H_0_ and presents a statistically significant difference between AB0 and AB10, AB20, and AB30 (*p* < 0.05).

The composite adhesive layer was evaluated using a MIRA 3 TESCAN GMX SE electron microscope (Tescan Brno s. r. o., Brno, Czech Republic), that is, the interaction at the filler/matrix interface and the bonded material/composite adhesive layer was evaluated. The microscopic samples were coated with gold using a Quorum Q150R ES device (Tescan Brno s. r. o., Brno, Czech Republic).

## 3. Results and Discussion

**Figure 4:**
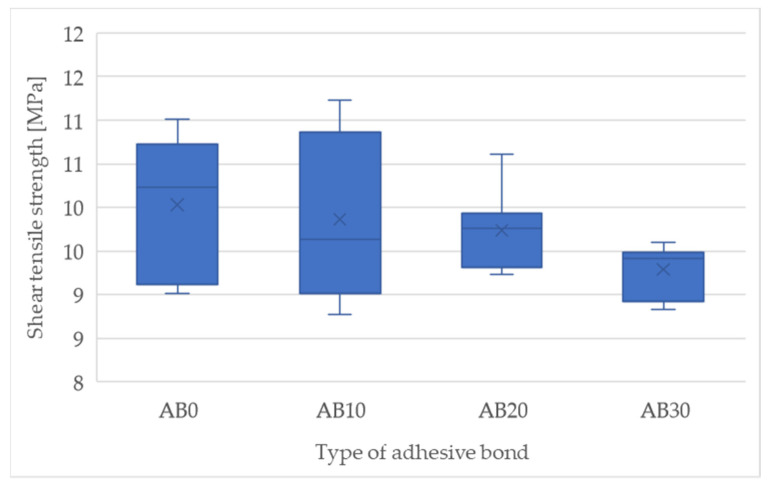
Shear tensile strength of adhesive bonds in static tensile test.

**Figure 5:**
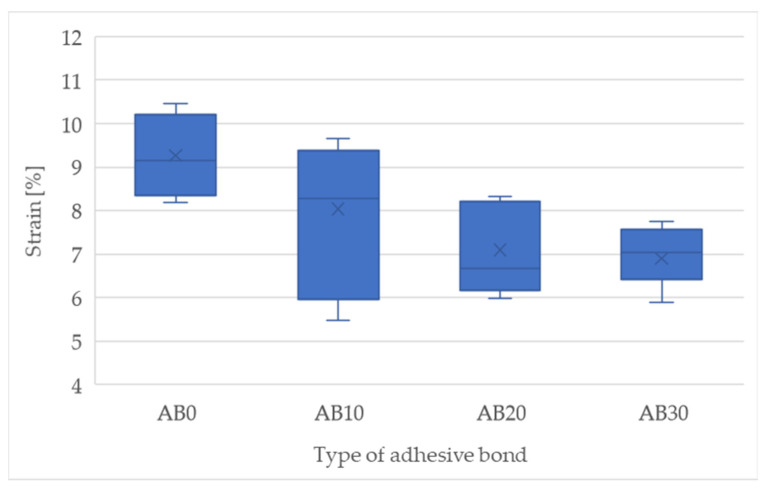
Strain of adhesive bonds in static tensile test.

The results of the mechanical properties based on the static shear test are shown in Figure 4 and Figure 5. Figure 4 presents the strength results of the adhesive bonds. The adhesive bond AB0 (etalon) showed a strength of 10.03 ± 0.71 MPa. With increasing filler concentration, the static strength of the adhesive bonds decreased. The adhesive bond AB10 showed a static strength of 9.86 ± 0.92 MPa (−1.70% compared to AB0), AB20 9.74 ± 0.43 MPa (−3%) and AB30 9.29 MPa (−8%). The results show a negative effect of increasing the filler concentration on the strength of the adhesive bonds. The fracture surface of the adhesive bonds AB0, AB10, AB20, and AB30 showed an adhesive-cohesive structure.

Statistical testing showed statistically insignificant differences between AB0 and AB10 (*p* = 0.72), AB20 (*p* = 0.40), that is, the filler concentration did not significantly affect the static strength of the AB10 and AB20 adhesive bonds. A statistically significant difference was demonstrated for the AB30 adhesive bond (*p* = 0.04), that is, the filler concentration significantly (negatively) affected the static strength of the AB30 adhesive bond.

The deformation of the adhesive bonds during the static test is shown in Figure 5. The adhesive bond AB0 showed a deformation of 9.27 ± 0.81%. With increasing filler concentration, that is, adhesive bonds AB10, AB20, and AB30, the deformation decreased. The AB10 adhesive bond showed a deformation of 8.04 ± 1.55% (1.5% vs. AB0), AB20 7.09 ± 0.89% (−31%), and AB30 6.90 ± 0.63% (−34%). The relatively high deformation of the adhesive bond AB0 means a reduced resistance to cyclic loading at higher intensities. This fact has been confirmed by other research [11,36].

Statistical testing showed a statistically insignificant difference between AB0 and AB10 (*p* = 0.11), that is, filler concentration did not significantly affect AB10 strain. A statistically significant difference was demonstrated for the adhesive bonds AB20 (*p* = 0.01) and AB30 (*p* = 0.01) adhesive bonds, that is, the filler concentration significantly affected the deformation of the adhesive bonds AB20 and AB30.

**Figure 6:**
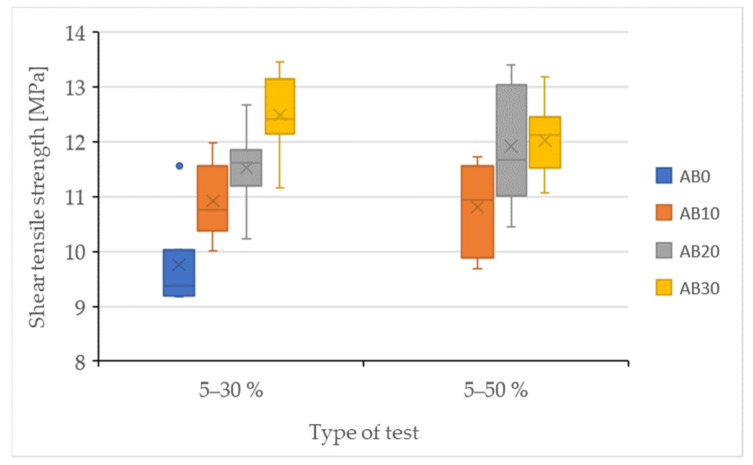
Results of the quasi-static test of adhesive bonds—strength.

**Figure 7:**
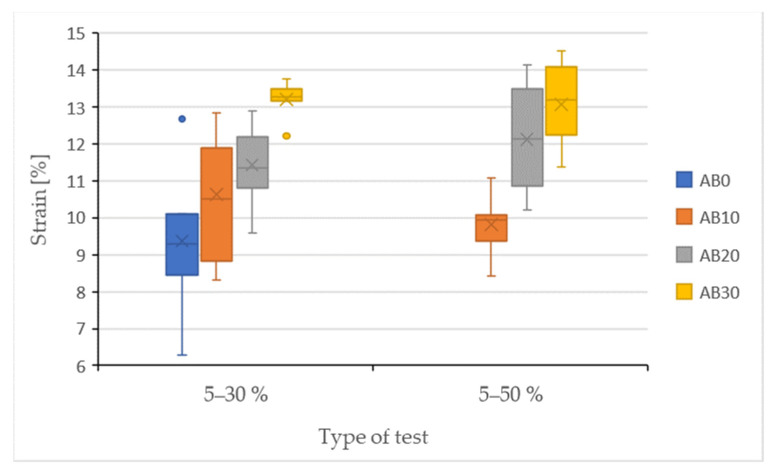
Results of the quasi-static test of adhesive bonds—strain.

The results of the quasi-static tests are shown in Figure 6 and Figure 7 and Table 3. The strength results of the adhesive bonds after the quasi-static test are shown in Figure 6. From the results in Figure 6 shows a decrease in the strength of the adhesive bond AB0 after a quasi-static test with a stress intensity between 5–30% (strength 9.76 ± 0.79 MPa) compared to the static test (strength 10.03 ± 0.71 MPa). It follows that the resulting strength of the adhesive bond AB0 was negatively affected by cyclic loading. The strength of the adhesive bonds under cyclic loading was positively affected by the addition of filler, that is, for the adhesive bonds AB10, AB20, and AB30. Figure 6 shows an increase in strength after a quasi-static test of 5–30% for all types of adhesive bonds with a composite adhesive layer, that is, AB10, AB20, and AB30. For adhesive bonds AB10 there was an increase in strength compared to AB0 by 10.67% to 10.92 ± 0.64 MPa, for AB20 by 15.34% to 11.53 ± 0.68 MPa, and AB30 by 21.88% to 12.49 ± 0.71 MPa. These results show that with increasing filler concentration, the strength of adhesive bonds increases in a quasi-static test with a lower stress intensity of 5–30%. Statistical testing of experiments with lower stress intensity of 5–30% showed statistically significant differences between the adhesive bond AB0 and AB10, AB20, and AB30 (*p* = 0.01), that is, the added filler significantly affected the strength of adhesive bonds with the composite adhesive layer. All types of adhesive bonds, that is, AB0, AB10, AB20, and AB30, withstood the specified number of 1000 cycles at cyclic loading with a lower intensity of 5–30%. Statistical testing of experiments with lower stress intensity of 5–30% showed statistically significant differences between the adhesive bond AB0 and AB10, AB20, and AB30 (*p* = 0.01), that is, the added filler significantly affected the strength of adhesive bonds with the composite adhesive layer. All types of adhesive bonds, that is, AB0, AB10, AB20, and AB30, withstood the specified number of 1000 cycles at cyclic loading with a lower intensity of 5–30%. Statistical testing of experiments with lower stress intensity of 5–30% showed statistically significant differences between the adhesive bond AB0 and AB10, AB20, and AB30 (*p* = 0.01), that is, the added filler significantly affected the strength of adhesive bonds with the composite adhesive layer. All types of adhesive bonds, that is, AB0, AB10, AB20, and AB30, withstood the specified number of 1000 cycles at cyclic loading with a lower intensity of 5–30%.

The AB0 adhesive bond did not withstand the specified 1000 cycles in a quasi-static test with a higher intensity of 5–50%. The average number of completed cycles was 241 ± 25 cycles. From this, the assumption that premature failure of the adhesive bond can occur even with a relatively low number of completed cycles follows and was confirmed [27]. Therefore, the service life of the AB0 adhesive bond was low. However, the added filler positively affected not only the mechanical properties, but also the service life of adhesive bonds at higher cyclic loading levels, because all adhesive bonds with composite adhesive layer, that is, AB10, AB20, and AB30, withstood the specified 1000 cycles, as shown in Table 4. Adhesive bond AB10 reached strength 10.81 ± 0.71 MPa, AB20 11.91 ± 1.01 MPa, and AB30 12.03 ± 0.63 MPa. Statistical testing could not be performed due to premature failure of the AB0 adhesive bond at 5–50%.

When comparing the strength between a quasi-static test with a lower intensity of 5–30% and a higher intensity of 5–50%, it is evident that at a higher intensity there was a slight decrease in strength compared to a lower intensity for adhesive bonds AB10 and AB30. AB10 adhesive bonds decreased by 1.07% to 11.91 ± 1.01 MPa. For adhesive bonds AB30 by 3.82% to 12.03 ± 0.63 MPa. Thus, the negative effect of a higher value of cyclic loading is evident, which causes a deterioration of the mechanical properties, that is, the strength of said adhesive bonds. However, the above conclusions about the decreasing in strength are not significant.

The results of strain after the quasi-static test are shown in Figure 7. The adhesive bond AB0 showed a strain of 9.27 ± 0.81% after the static test. After a 5–30% quasi-static test, the AB0 adhesive bond showed a slight increase to 9.37 ± 1.80%. Adhesive bonds AB10, AB20, and AB30 showed a significant increase in strains compared to AB0 after a quasi-static test of 5–30%. Adhesive bonds AB10 10.63 ± 1.51%, AB20 11.43 ± 0.99%, and AB30 13.20 ± 0.44%. Therefore, the percentage increase was approximately from 12 to 29%. Statistical testing showed statistically insignificant differences between AB0 and AB10 (*p* = 0.21), that is, the filler concentration did not significantly affect the strain of the AB10 adhesive bond. A statistically significant difference was demonstrated for AB20 (*p* = 0.01) and AB30 (*p* = 0.01) adhesive bonds, that is, the filler concentration significantly affected the strain of said adhesive bonds.

The AB0 adhesive bond did not withstand the specified 1000 cycles in a quasi-static test with a higher intensity of 5–50%. For this reason, the strain could not be quantified. For adhesive bonds AB10 the strain was 9.82 ± 0.74 MPa, for AB20 12.12 ± 1.30% and for AB30 13.07 ± 0.99%. Statistical testing could not be performed due to premature failure of the AB0 adhesive bond at 5–50%.

From Table 3 it can be seen that almost all variants of the adhesive bonds have withstood the specified number of 1000 cycles. The exception occurred only for the adhesive bond AB0 in the test 5–50%, when the stated intensity of cyclic loading could not withstand any adhesive bond, that is, the number of completed cycles was 241 ± 25. From the above result it is possible to observe the fact that the adhesive bond AB0 is not suitable for applications, where higher values of cyclic loading occur. At a higher value of cyclic loading, the effect of the added filler had a positive effect, that is, adhesive bonds AB10, AB20, and AB30, when all adhesive bonds lasted a specified number of 1000 of cycles. The service life of the mentioned adhesive bonds thus increased significantly compared to the AB0 adhesive bond at an intensity of 5–50%. Broughton et al. [39] in their report state that the upper limit of 50% of the static test is used in the aerospace industry to determine safety factors in the design of bonded and bolted structures under cyclic loading.

Table 4 shows the evaluation of the type of failure of individual types of adhesive bonds, and it is evident that the adhesive bonds AB0 showed the type of adhesion-cohesion failure in all types of tests. One adhesive bond showed an adhesive failure of 5–30% after a quasi-static test, which could be caused by insufficient preparation of the bonded surface. AB10 adhesive bonds showed adhesion and adhesion-cohesion type failure after static test, adhesion type failure after quasi-static test with intensity 5–30% and mostly adhesion failure after quasi-static test with intensity 5–50%. These types of failures were similar for all types of adhesive bonds with a composite adhesive layer. The adhesive type of failure means a reduced adhesion of the composite adhesive layer to the bonded material. The low adhesion of the composite adhesive layer could be caused by insufficient preparation of the bonded material, an undesirable layer on the surface of the filler, e.g., grease, which affects the whole structure of the composite layer and, thus, the adhesion to the bonded material. This problem can be solved by a suitable surface treatment of the natural filler [40,41,42]. Another reason for the occurrence of adhesive failure in bonds with a composite adhesive layer, such as AB10, AB20, and AB30, could be the increased thickness of the adhesive layer compared to adhesive bonds AB0, as shown in Table 2, which showed an adhesion-cohesion type of failure. The increased thickness of the adhesive layer causes an increase in the tensile bending moment, and, thus, a decrease in the adhesion at the adhesive/adherend interface. Grant et al. [43] verified that as the thickness of the bonded layer increases, the bending stress increases as the bending moment increases. As a result, the strength of the adhesive bond also decreases. Da Silva et al. [44] found that the optimal thickness of the bonded layer in terms of mechanical properties is up to 0.5 mm.

**Figure 8:**
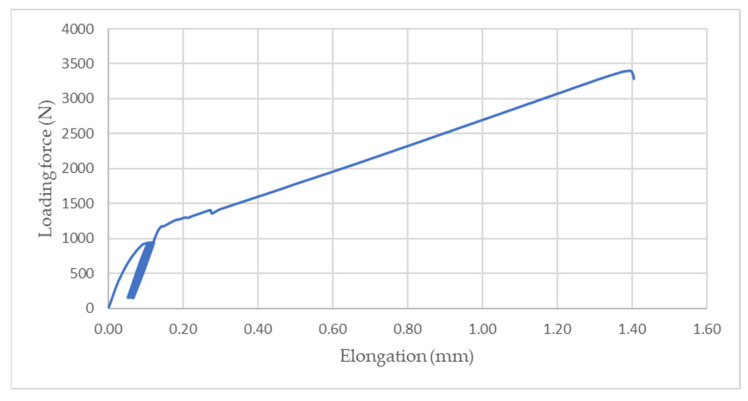
Quasi-static curve of adhesive bond AB10 at cyclic loading intensity 5–30%.

**Figure 9:**
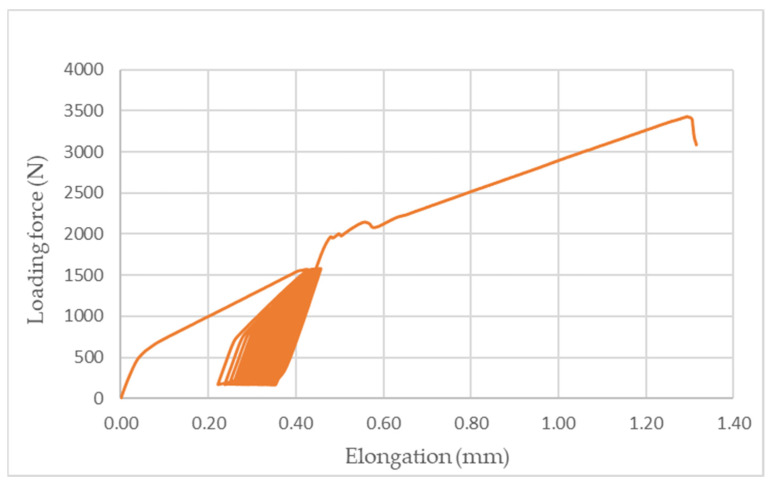
Quasi-static curve of AB20 adhesive bond at cyclic loading intensity 5–50%.

**Figure 10:**
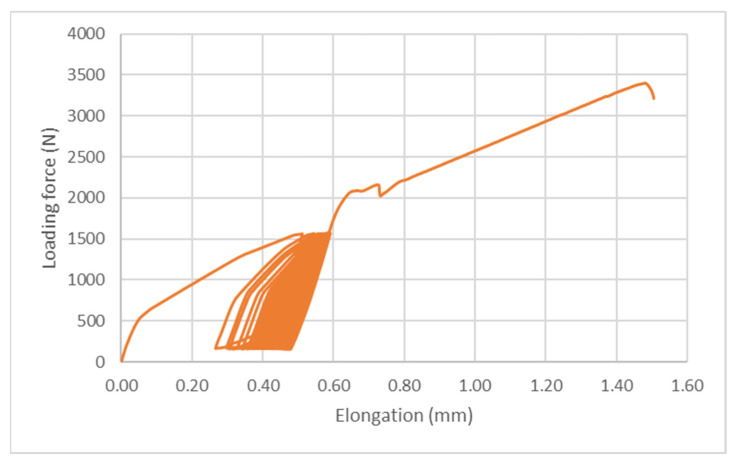
Quasi-static curve of AB30 adhesive bond at cyclic loading intensities of 5–30% and 5–50%.

Figure 8, Figure 9 and Figure 10 demonstrate an example of quasi-static curves that present the course of stress of adhesive bonds AB10, AB20, and AB30 at different cyclic loading intensities, that is, 5–30% and 5–50%. Figures also show the intensity of viscoelastic behavior. From Figure 8 it can be seen that for the adhesive bond AB10 the elongation at the cyclic loading intensity was smaller than at the cyclic loading intensity of 5–50%, as shown in Figure 9 and Figure 10. Gradual elongation of the adhesive bond under cyclic loading means gradual bond fatigue. These characteristics have been demonstrated for all types of adhesive bonds. It follows that the higher the intensity of cyclic loading, the sooner the bond will fail, that is, the mechanical properties and service life of the adhesive bonds are adversely affected, as demonstrated by research that has addressed higher intensities of cyclic loading up to 70% of maximum forces [10,11,36].

The results of the experiments confirmed the positive effect of the filler in the form of coconut shell particles on the mechanical properties and service life of adhesive bonds under cyclic loading. In the study by Shahar et al. [45], they state that the natural filler of kenaf in the form of small particles in the polymer matrix positively affects its fatigue life, due to the better stress distribution in the matrix and the ability to form good bonds at the filler/matrix interface. Abdullah et al. [46] reached similar results.

**Figure 11:**
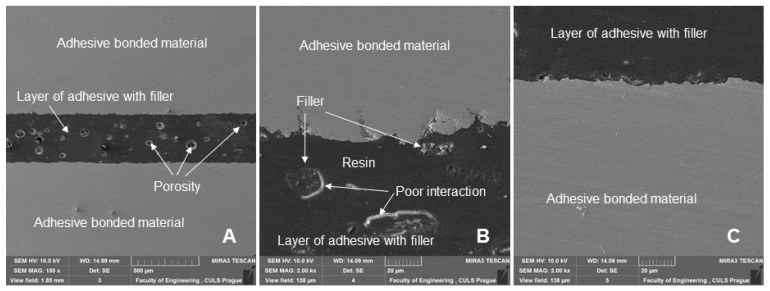
SEM images of an adhesive bond with a biological filler based on coconut microparticles—designation AB20, 0 cycles: (**A**): adhesive bond (MAG 100 ×), (**B**): adhesive bond with a detailed view of the adhesive layer and the interaction of the filler with resin (MAG 2.00 k×), (**C**): section of the adhesive bond with a detailed view of the adhesive layer of the adhesive bond (MAG 2.00 k×).

**Figure 12:**
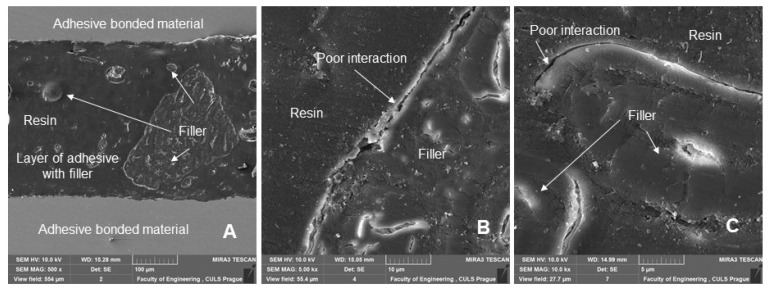
SEM images of an adhesive bond section with a biological filler based on coconut microparticles—designation AB10, quasi-static test from 5 to 30% (157–940 N), 1000 cycles: (**A**): adhesive bond section (MAG 500 ×), (**B**): section of the adhesive bond with a detailed view of the interaction of filler and resin (MAG 5.00 k×), (**C**): section of the adhesive bond with a detailed view of the interaction of filler and resin (MAG 10.00 k×).

**Figure 13:**
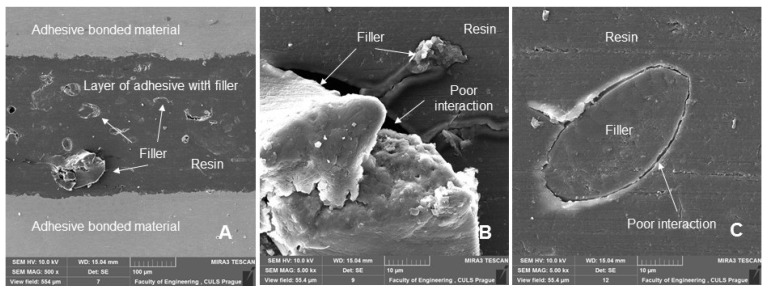
SEM images of an adhesive bond section with a biological filler based on coconut microparticles—designation AB20, quasi-static test from 5 to 50% (157–1567 N), 1000 cycles: (**A**): adhesive bond section (MAG 500 ×), (**B**): section of the adhesive bond with a detailed view of the interaction of filler and resin (MAG 5.00 k×), (**C**): section of the adhesive bond with a detailed view of the interaction of filler and resin (MAG 5.00 k×).

**Figure 14:**
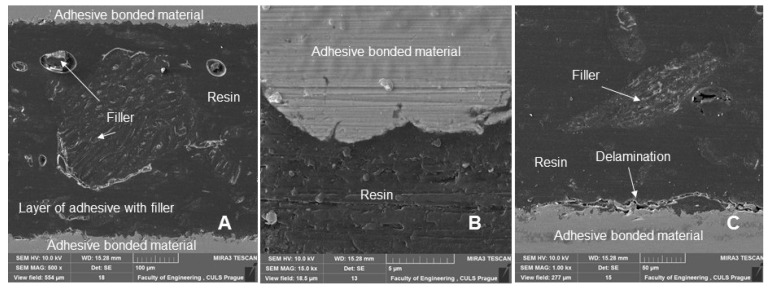
SEM images of an adhesive bond section with a biological filler based on coconut microparticles—designation AB30, quasi-static test from 5 to 50% (157–1567 N), 1000 cycles: (**A**): adhesive bond section (MAG 500 ×), (**B**): section of the adhesive bond with a detailed view of the adhesive layer and the interaction of the bonded material and the composite bonded layer (MAG 15.00 k×), (**C**): section of the adhesive bond with a detailed view of the adhesive layer and the interaction of the bonded material and the composite bonded layer with significant delamination (MAG 1.00 k×).

Figure 11 shows a section of an adhesive bond with a filler weight concentration of 20% of microparticles of crushed coconut shells (designated AB20), which were not exposed to cyclic loading of the adhesive bond, that is, 0 cycles. Figure 11A shows an overview picture presenting a section of the adhesive bond. Figure 11A shows the adhesive and composite cohesive layer of the adhesive bond. Figure 11A shows the porosity inside the adhesive layer. Figure 11B,C show a good interaction between the adhesive layer and the bonded material. Figure 11B shows a low interaction (wettability) between the filler and the resin. This negative phenomenon can also be observed in the other sections of the adhesive bonds shown in Figure 12, Figure 13 and Figure 14. The reduced wettability between the filler and matrix could be due to the coconut oil content of the particulate filler. Hasanah et al. [47] and Gao et al. [48] obtained coconut shell oil by pyrolysis process, which clearly indicates the presence of oil in the coconut shell. This factor could be improved, for example, by chemical treatment of the filler [41].

Figure 12 shows a section of an adhesive bond with a filler concentration of 10% of microparticles of crushed coconut shells (designated AB10), which were subjected to a quasi-static test from 5 to 30% (157–940 N) for 1000 cycles. Figure 12 shows the distribution of the filler, as well as its size and geometric shape. Figure 12B,C show a detailed view of the filler, which was characterized by considerable shape variability and reduced degree of wetting. 

Figure 13 shows a section of an adhesive bond with a filler concentration of 20% of microparticles of crushed coconut shells (designated AB20), which were subjected to a quasi-static test from 5 to 50% (157–1567 N) for 1000 cycles. Figure 13 again shows the distribution of the filler, as well as its size and geometric shape. Figure 13B,C show a detailed view of the filler, which was characterized by considerable shape variability seen from a comparison of the two figures and a reduced rate of wettability. The interaction was particularly poor for irregular particles. This phenomenon was identified in the research of activated rubber particles in polymer composite materials [49].

Figure 14 shows a section of an adhesive bond with a filler concentration of 30% of microparticles of crushed coconut shells (designated AB30), which were subjected to a quasi-static test from 5 to 50% (157–1567 N) for 1000 cycles. Figure 14 again shows the distribution of the filler and its significant difference in the size of the filler and its geometric shape. Figure 14B does not show damage to the adhesive bonds between the bonded material and the composite adhesive layer even after 1000 cycles. This delamination caused by cyclic loading is evident from Figure 14C. This is a single cut with an adhesive bond. This delamination leads to the initiation of the adhesive-cohesive fracture surface at the interface of the bonded layer between the adhesive and the bonded material, which is clearly seen in Figure 14C. The delamination is visible in the lower part of the figure by a longitudinal section of the adhesive bond (Figure 14C).

## 4. Conclusions

The research follows the current trend in the field of polymer composites are fillers based on natural material (or waste). The material utilization of natural waste as filler sets the current trend in the research of polymer matrix based composite materials. By modifying the adhesives with a natural filler based on particles or fibers, a composite layer can be obtained which can improve the parameters of the adhesive bond in terms of mechanical properties, increased service life, and, thus, increased safety under cyclic loading. The results of experiments of adhesive bonds with a composite layer of glue based on natural waste from the process of processing coconut oil, stressed by cyclic loading (quasi-static tests) showed:

- The thickness of the adhesive layer was AB0 = 31± 4 μm, AB10 = 349 ± 6 μm, AB20 = 303 ± 6 μm, AB30 = 464 ± 8 μm;
- The results of the static shear test showed a reduction in shear strength for all types of adhesive bonds with a composite adhesive layer, that is, AB10, AB20, and AB30, in the range from 1.7 to 8% compared to an adhesive bond without AB0 filler. Similar results were achieved by strain, where the decrease ranged from 15 to 34% compared to AB0. Statistical testing showed statistically insignificant differences between AB0 and AB10 (*p* = 0.72), AB20 (*p* = 0.40) and a statistically significant difference between AB0 and AB30 (*p* = 0.04);
- Adhesive bonds without AB0 filler showed approximately 2.8% less strength after the quasi-static 5–30% test compared to the static test. The adhesive bonds with the composite adhesive layer, that is, AB10, AB20, and AB30, showed 5–30% higher strength in the quasi-static tests in the range from 11 to 22% compared to AB0. The strains increased by 12 to 29%. Statistical testing of experiments with lower stress intensity of 5–30% showed statistically significant differences between the adhesive bond AB0 and AB10, AB20, and AB30 (*p* = 0.01);
- AB0 adhesive bonds did not withstand the specified 1000 cycles in a quasi-static test with an intensity of 5–50%, that is, service life was negatively affected by cyclic loading of higher intensity. Adhesive bonds with a composite adhesive layer, that is, AB10, AB20, and AB30, lasted the specified 1000 cycles. Therefore, the added filler had a positive effect on the service life of the adhesive bonds with the composite adhesive layer under the cyclic loading;
- The experimental results showed an optimum filler concentration of 20 wt.% in terms of cyclic loading intensity, that is, 5–30% and 5–50%. This concentration of filler showed an increase in strength in cyclic tests of 5–50% intensity compared with 5–30% intensity. Therefore, it can be said that not only the durability was increased but also the mechanical properties were improved. For other types of adhesive bonds with composite adhesive layer, that is, AB10 and AB30, there is a decrease in mechanical properties at higher cyclic stress intensity. SEM analysis showed reduced wettability of the filler and matrix, which was probably due to the coconut oil contained in the coconut shell filler. Considerable shape variability of the particulate filler was demonstrated. Delamination caused by cyclic stresses of 5–50% was demonstrated in the AB30 bonded joint, which led to the initiation of an adhesive/cohesive fracture at the interface between the adhesive layer and the bonded material.

## Figures and Tables

**Table 1:** Basic mechanical properties of steel S235J0 at a temperature of 20 °C [37].

Tensile Strength	340–470 MPa
Yield Strength	225–235 MPa
Elastic Modulus	212 GPa
Elongation	24%

**Table 2:** Type of adhesive bonds and their characteristics.

Type of Adhesive Bond	Bonded Layer Thickness (μm)	Characteristics
AB0	31 ± 4	Adhesive bond with adhesive without filler
AB10	349 ± 6	Adhesive bond with filler concentration of 10% by weight
AB20	303 ± 6	Adhesive bond with filler concentration of 20% by weight
AB30	464 ± 8	Adhesive bond with filler concentration of 30% by weight

**Table 3:** Evaluation of cyclic tests of adhesive bonds.

Type of Adhesive Bond	Type of Test	Number of Test Samples (Number of Finished Tests/Total Number of Tests)	Number of Finished Cycles
AB0	from 5 to 30% (157–940 N)	7/7	1000
	from 5 to 50% (157–1567 N)	0/7	241 ± 25
AB10	from 5 to 30% (157–940 N)	7/7	1000
	from 5 to 50% (157–1567 N)	7/7	1000
AB20	from 5 to 30% (157–940 N)	7/7	1000
	from 5 to 50% (157–1567 N)	7/7	1000
AB30	from 5 to 30% (157–940 N)	7/7	1000
	from 5 to 50% (157–1567 N)	7/7	1000

**Table 4:** Evaluation of the type of failure of individual types of adhesive bonds.

Type of Adhesive Bond	Characteristics of the Adhesive Bond Test	AF ^1^	A/CF ^2^
AB0	Static test	0/7	7/7
	Quasi-static test from 5 to 30% (157–940 N)	1/7	6/7
	Quasi-static test from 5 to 50% (157–1567 N)	0/7	7/7
AB10	Static test	3/7	4/7
	Quasi-static test from 5 to 30% (157–940 N)	7/7	0/7
	Quasi-static test from 5 to 50% (157–1567 N)	5/7	2/7
AB20	Static test	1/7	6/7
	Quasi-static test from 5 to 30% (157–940 N)	6/7	1/7
	Quasi-static test from 5 to 50% (157–1567 N)	6/7	1/7
AB30	Static test	0/7	7/7
	Quasi-static test from 5 to 30% (157–940 N)	4/7	3/7
	Quasi-static test from 5 to 50% (157–1567 N)	5/7	2/7

^1^ Adhesive failure; ^2^ Adhesive/cohesive failure.

## Data Availability

Not applicable.

## References

[1] Lapique F., Redford K. (2002). Curing effects on viscosity and mechanical properties of a commercial epoxy resin adhesive. Int. J. Adhes. Adhes..

[2] Bhowmik S., Bonin H., Bui V., Weir R. (2006). Durability of adhesive bonding of titanium in radiation and aerospace environments. Int. J. Adhes. Adhes..

[3] Barnes T., Pashby I. (2000). Joining techniques for aluminium spaceframes used in automobiles: Part II—Adhesive bonding and mechanical fasteners. J. Mater. Process. Technol..

[4] Preu H., Mengel M. (2007). Experimental and theoretical study of a fast curing adhesive. Int. J. Adhes. Adhes..

[5] Adams R. (2005). Adhesive Bonding: Science, Technology and Applications.

[6] Pizzi A., Mittal K. (2003). Handbook of Adhesive Technology.

[7] Saraç İ., Adin H., Temiz Ş. (2018). Experimental determination of the static and fatigue strength of the adhesive joints bonded by epoxy adhesive including different particles. Compos. Part B Eng..

[8] Yan L., Kasal B., Huang L. (2016). A review of recent research on the use of cellulosic fibres, their fibre fabric reinforced cementitious, geo-polymer and polymer composites in civil engineering. Compos. Part B Eng..

[9] Lau K.T., Hung P.Y., Zhu M.H., Hui D. (2018). Properties of natural fibre composites for structural engineering applications. Compos. Part B Eng..

[10] Müller M., Valášek P., Kolář V., Šleger V., Gürdil G.A.K., Hromasová M., Hloch S., Moravec J., Pexa M. (2019). Material Utilization of Cotton Post-Harvest Line Residues in Polymeric Composites. Polymers.

[11] Kolář V., Müller M., Mishra R., Rudawska A., Šleger V., Tichý M., Hromasová M., Valášek P. (2020). Quasi-Static Tests of Hybrid Adhesive Bonds Based on Biological Reinforcement in the Form of Eggshell Microparticles. Polymers.

[12] Sareena C., Ramesan M., Purushothaman E. (2012). Utilization of coconut shell powder as a novel filler in natural rubber. J. Reinf. Plast. Compos..

[13] Kumar M., Jena H. Sea Shell: A Marine Waste to Filler in Polymer Composite. Proceedings of the International Conference on Artificial Intelligence in Manufacturing & Renewable Energy (ICAIMRE) 2019.

[14] Olumuyiwa A.J., Isaac T.S., Samuel S.O. (2012). Study of Mechanical Behaviour of Coconut Shell Reinforced Polymer Matrix Composite. J. Miner. Mater. Charact. Eng..

[15] Keerthika B., Umayavalli M., Jeyalalitha T., Krishnaveni N. (2016). Coconut shell powder as cost effective filler in copolymer of acrylonitrile and butadiene rubber. Ecotoxicol. Environ. Saf..

[16] Orue A., Eceiza A., Arbelaiz A. (2020). The use of alkali treated walnut shells as filler in plasticized poly(lactic acid) matrix composites. Ind. Crop. Prod..

[17] Ramnath B.V., Jeykrishnan J., Ramakrishnan G., Barath B., Ejoelavendhan E., Raghav P.A. (2018). Sea Shells And Natural Fibres Composites: A Review. Proceedings of the Materials Today: Proceedings.

[18] Müller M., Valášek P. (2018). Composite adhesive bonds reinforced with microparticle filler based on egg shell waste. J. Phys. Conf. Ser..

[19] Bahrami B., Ayatollahi M., Beigrezaee M., da Silva L. (2019). Strength improvement in single lap adhesive joints by notching the adherends. Int. J. Adhes. Adhes..

[20] Stoeckel F., Konnerth J., Gindl-Altmutter W. (2013). Mechanical properties of adhesives for bonding wood—A review. Int. J. Adhes. Adhes..

[21] Kolář V., Tichý M., Müller M., Valášek P., Rudawska A. (2019). Research on influence of cyclic degradation process on changes of structural adhesive bonds mechanical properties. Agron. Res..

[22] Han X., Crocombe A., Anwar S., Hu P. (2014). The strength prediction of adhesive single lap joints exposed to long term loading in a hostile environment. Int. J. Adhes. Adhes..

[23] Krolczyk G., Raos P., Legutko S. (2014). Experimental analysis of surface roughness and surface texture of machined and fused deposition modelled parts. Teh. Vjesn..

[24] Nieslony P., Krolczyk G., Wojciechowski S., Chudy R., Żak K., Maruda R. (2018). Surface quality and topographic inspection of variable compliance part after precise turning. Appl. Surf. Sci..

[25] Rudawska A. (2014). Selected aspects of the effect of mechanical treatment on surface roughness and adhesive joint strength of steel sheets. Int. J. Adhes. Adhes..

[26] Bresson G., Jumel J., Shanahan M.E., Serin P. (2012). Strength of adhesively bonded joints under mixed axial and shear loading. Int. J. Adhes. Adhes..

[27] Broughton W.R., Mera R.D., Hinopoulos G. (1999). Cyclic Fatigue Testing of Adhesive Joints Test Method Assessment.

[28] Chen Y., Smith L.V. (2021). Ratcheting and recovery of adhesively bonded joints under tensile cyclic loading. Mech. Time-Dependent Mater..

[29] Zhang J., Li H., Li H.-Y., Wei X.-L. (2019). Uniaxial ratchetting and low-cycle fatigue failure behaviors of adhesively bonded butt-joints under cyclic tension deformation. Int. J. Adhes. Adhes..

[30] Naebe M., Abolhasani M.M., Khayyam H., Amini A., Fox B. (2016). Crack Damage in Polymers and Composites: A Review. Polym. Rev..

[31] Xia Z., Shen X., Ellyin F. (2005). Biaxial cyclic deformation of an epoxy resin: Experiments and constitutive modeling. J. Mater. Sci..

[32] Tao G., Xia Z. (2007). Ratcheting behavior of an epoxy polymer and its effect on fatigue life. Polym. Test..

[33] Arena N., Lee J., Clift R. (2016). Life Cycle Assessment of activated carbon production from coconut shells. J. Clean. Prod..

[34] Faisal Z.H.T., Amri F., Tahir I. (2010). Effect of Maleic Anhydride Polypropylene on the Properties of Coconut Shell Filled Thermoplastic Elastomeric Olefin Composites. Indones. J. Chem..

[35] International Organization for Standardization (2009). ČSN EN 1465—Adhesives—Determination of Tensile Lap-Shear Strength of Bonded Assemblies.

[36] Tichý M., Kolář V., Müller M., Mishra R.K., Šleger V., Hromasová M. (2020). Quasi-Static Shear Test of Hybrid Adhesive Bonds Based on Treated Cotton-Epoxy Resin Layer. Polymers.

[37] DIN 17120 Grade St 37-3—Low Carbon Steel—Matmatch. https://matmatch.com/materials/minfm31305-din-17120-grade-st-37-3.

[38] (1995). Adhesives—Designation of Main Failure Patterns.

[39] Broughton W.R., Mera R.D. (1999). Cyclic Fatigue Testing of Adhesive Joints Environmental Effects.

[40] Aziz S.H., Ansell M.P. (2004). The effect of alkalization and fibre alignment on the mechanical and thermal properties of kenaf and hemp bast fibre composites: Part 1—Polyester resin matrix. Compos. Sci. Technol..

[41] Valášek P., Müller M., Šleger V., Kolář V., Hromasová M., D’Amato R., Ruggiero A. (2021). Influence of Alkali Treatment on the Microstructure and Mechanical Properties of Coir and Abaca Fibers. Materials.

[42] Cai M., Takagi H., Nakagaito A.N., Katoh M., Ueki T., Waterhouse G.I., Li Y. (2015). Influence of alkali treatment on internal microstructure and tensile properties of abaca fibers. Ind. Crop. Prod..

[43] Grant L., Adams R., da Silva L.F. (2009). Experimental and numerical analysis of single-lap joints for the automotive industry. Int. J. Adhes. Adhes..

[44] Da Silva L.F., Carbas R., Critchlow G., Figueiredo M., Brown K. (2009). Effect of material, geometry, surface treatment and environment on the shear strength of single lap joints. Int. J. Adhes. Adhes..

[45] Shahar F.S., Sultan M.T.H., Safri S.N.A., Jawaid M., Abu Talib A.R., Basri A.A., Shah A.U.M. (2021). Fatigue and impact properties of 3D printed PLA reinforced with kenaf particles. J. Mater. Res. Technol..

[46] Abdullah A.H., Alias S.K., Abdan K., Ali A. (2012). A study of fatigue life of kenaf fibre composites. Proceedings of the Advanced Materials Research.

[47] Hasanah U., Setiaji B., Triyono T., Anwar C. (2012). The Chemical Composition and Physical Properties of the Light and Heavy Tar Resulted from Coconut Shell Pyrolysis. J. Pure Appl. Chem. Res..

[48] Gao Y., Yang Y., Qin Z., Sun Y. (2016). Factors affecting the yield of bio-oil from the pyrolysis of coconut shell. SpringerPlus.

[49] Müller M., Valášek P., Rudawska A., Choteborsky R. (2018). Effect of active rubber powder on structural two-component epoxy resin and its mechanical properties. J. Adhes. Sci. Technol..

